# Pathway Enrichment Analysis of Whole-Exome Sequencing Data from Formalin-Fixed, Paraffin-Embedded Enucleated Eyes with Retinoblastoma and Choroidal Malignant Melanoma

**DOI:** 10.7759/cureus.110548

**Published:** 2026-06-09

**Authors:** Toshihiko Matsuo, Takehiro Tanaka, Daisuke Ennishi, Akira Saito, Mitsuhiro Amemiya, Shigeo Kamitsuji

**Affiliations:** 1 Ophthalmology, Graduate School of Interdisciplinary Science and Engineering in Health Systems, Okayama University, Okayama City, JPN; 2 Pathology, Graduate School of Medicine, Dentistry, and Pharmaceutical Sciences, Okayama University, Okayama, JPN; 3 Medical Oncology, Graduate School of Medicine, Dentistry, and Pharmaceutical Sciences, Okayama University, Okayama City, JPN; 4 Genomic Statistics, Stagen Co. Ltd., Tokyo, JPN

**Keywords:** choroidal malignant melanoma, driver genes (driver mutations), enucleation, formalin-fixed paraffin-embedded (ffpe), integrative oncogenomics, pathway enrichment, retinoblastoma, somatic mutation, tohoku medical megabank, whole-exome sequencing

## Abstract

Objectives: Intraocular tumors are extremely rare, small in size, and difficult to approach by biopsy. In the era of cancer genome analysis, we designed a pilot study to perform whole-exome sequencing of formalin-fixed paraffin-embedded enucleated eyes of retinoblastoma and choroidal malignant melanoma as two major intraocular malignancies.

Methodology: Genomic DNA was isolated from intraocular tumor areas of 105 paraffin sections with a 5 μm thickness of seven enucleated eyes with retinoblastoma and seven eyes with choroidal malignant melanoma. One of 7 samples of retinoblastoma and another of seven samples of choroidal malignant melanoma were excluded from the study since the sequencing output and depth of reads were lower compared with the other samples. The sequencing data after quality control were aligned to the reference genome sequence (hg38, GRCh38 Assembly, Genome Reference Consortium Human Build 38), and the mapped reads were processed to improve data quality. Somatic mutations (single nucleotide variants, insertions and deletions, and multiple nucleotide variants) in each sample were extracted after excluding variants reported in a Panel of Normals (PON) from the 1000 Genomes Project. Additional selection criteria included a mutation depth of ≥5 reads and either no registration in or an allele frequency of less than 5% in the Tohoku Medical Megabank of Japan (ToMMo 60KJPN-SNV/INDEL Allele Frequency Panel).

Results: Candidate genes with somatic mutations were selected by three criteria: genes with the same mutation shared by two samples or more, recurrently mutated genes three times or over, and genes of driver candidates identified in combining several different driver mutation-detecting programs by Integrative OncoGenomics (IntOGen). Using candidate genes detected by any of the three criteria as input, enrichment analyses identified 28 pathways in Gene Ontology (GO) and 2 pathways in the Kyoto Encyclopedia of Genes and Genomes (KEGG) for retinoblastoma, while 385 pathways in GO, 12 in KEGG, 2 in the Hallmark gene set of the Molecular Signatures Database (MSigDB), and 47 in Reactome were identified for choroidal malignant melanoma. The enrichment maps showed three major pathways differently in retinoblastoma and choroidal malignant melanoma: one with dynein in retinoblastoma and another with *MET* in choroidal malignant melanoma.

Conclusions: Although there were limitations related to the small amounts of DNA available from formalin-fixed, paraffin-embedded small-sized tissues and the absence of matched normal control tissue, whole-exome sequencing provided clues to somatic mutations that were enriched in specific pathways and differed between retinoblastoma and choroidal malignant melanoma.

## Introduction

Intraocular tumors, including primary malignancies and metastatic cancers, are extremely rare in the field of ophthalmology. Two well-known primary intraocular tumors are retinoblastoma in children [1] and choroidal malignant melanoma in adults [2,3,4,5]. In addition, primary intraocular lymphoma, often in association with primary central nervous system lymphoma, is the third entity of primary intraocular tumors in ophthalmic practice [6,7,8,9]. These primary intraocular tumors give diagnostic challenges because of the difficulty in reaching a pathological diagnosis by biopsy, which is a common procedure in other malignancies. The diagnoses of intraocular tumors are based on clinical pictures observed by ocular fundus examinations, as well as computed tomography scans and magnetic resonance imaging. As for the treatment, resection of the tumor generally indicates the extirpation of the whole eyeball, called enucleation, which naturally leads to complete loss of vision. Therefore, the therapeutic decision whether to enucleate the eye with a tumor is carefully made according to risk and benefit considerations.

Cancer genome analysis of formalin-fixed, paraffin-embedded resected tissues by targeted sequencing panels (multigene panels) has now become a standard diagnostic procedure for identifying applicable therapeutic regimens based on mutated genes in unresectable and relapsed malignancies [10]. The cancer gene panels in clinical applications are targeted-exome sequencing in a limited number of genes, which picks up evidence-based candidate genes in cancers. Recently, targeted-exome sequencing with multigene panels as well as whole-exome sequencing have been tested to search for overall gene mutations in retinoblastoma [11,12,13,14,15,16,17,18]. In contrast, there is no study to perform whole-exome sequencing in choroidal malignant melanoma. Of course, in the other field of medicine, there have been many studies to apply whole-exome sequencing to analyze mucosal and cutaneous malignant melanoma [19,20,21,22,23,24].

Intraocular tumors, such as retinoblastoma and choroidal malignant melanoma, in formalin-fixed, paraffin-embedded enucleated eyes are small in size and have too little normal intraocular tissue for comparison. With these limitations in mind, we conducted a pilot study of whole-exome sequencing of retinoblastoma and choroidal malignant melanoma in formalin-fixed, paraffin-embedded enucleated eyes with a small sample size. The primary aim of this study was to determine whether whole-exome sequencing could be successfully performed in the two major intraocular tumors, retinoblastoma and choroidal malignant melanoma, and the secondary aim was to identify driver mutations in each malignancy for pathway enrichment analysis.

## Materials and methods

Patients, genomic DNA extraction, and whole-exome sequencing

**Figure 1:**
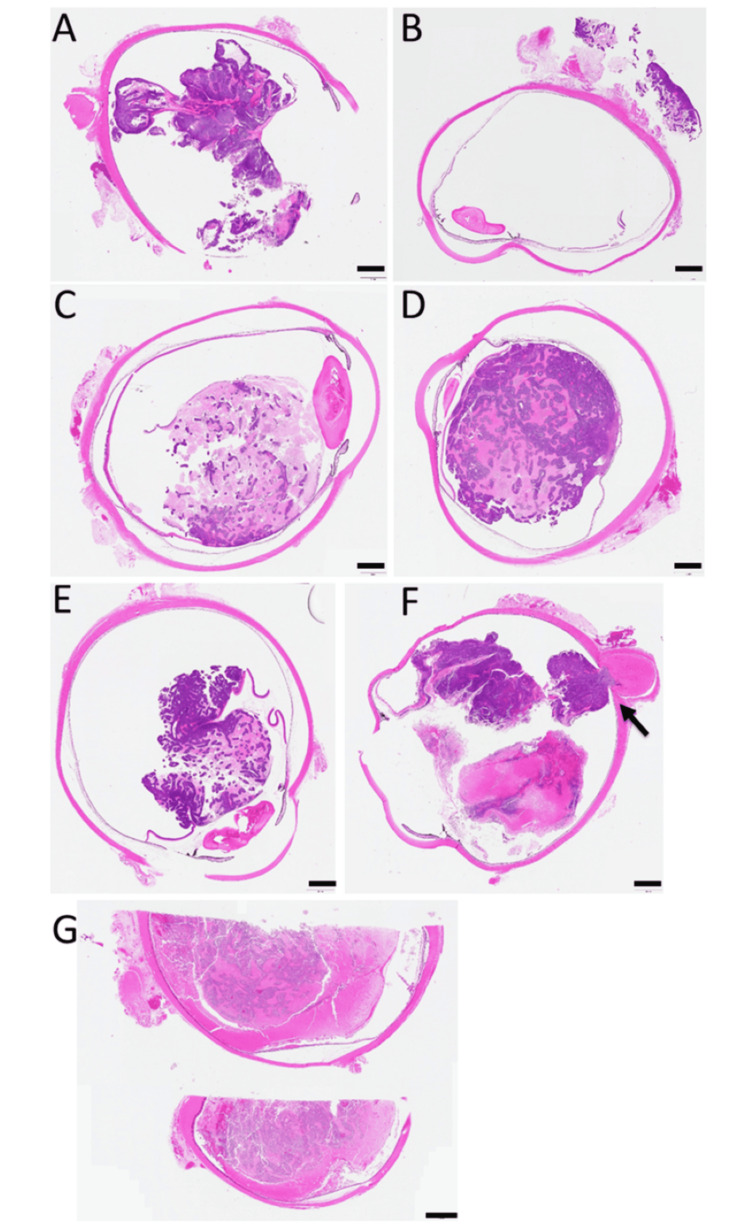
Hematoxylin-eosin-stained pathological sections of enucleated eyes with retinoblastoma. A: Case R1, B: Case R2, C: Case R3, D: Case R4, E: Case R6, F: Case R7, and G: Case R9. Note the tumor tissue outside the eyeball in Case R2, which is attributable to processing artifacts. Also note tumor infiltration into the optic nerve (arrow in F) in Case R7. This patient (R7) was the only patient who underwent prophylactic chemotherapy (Table 1). Scale bar = 1 mm.

**Figure 2:**
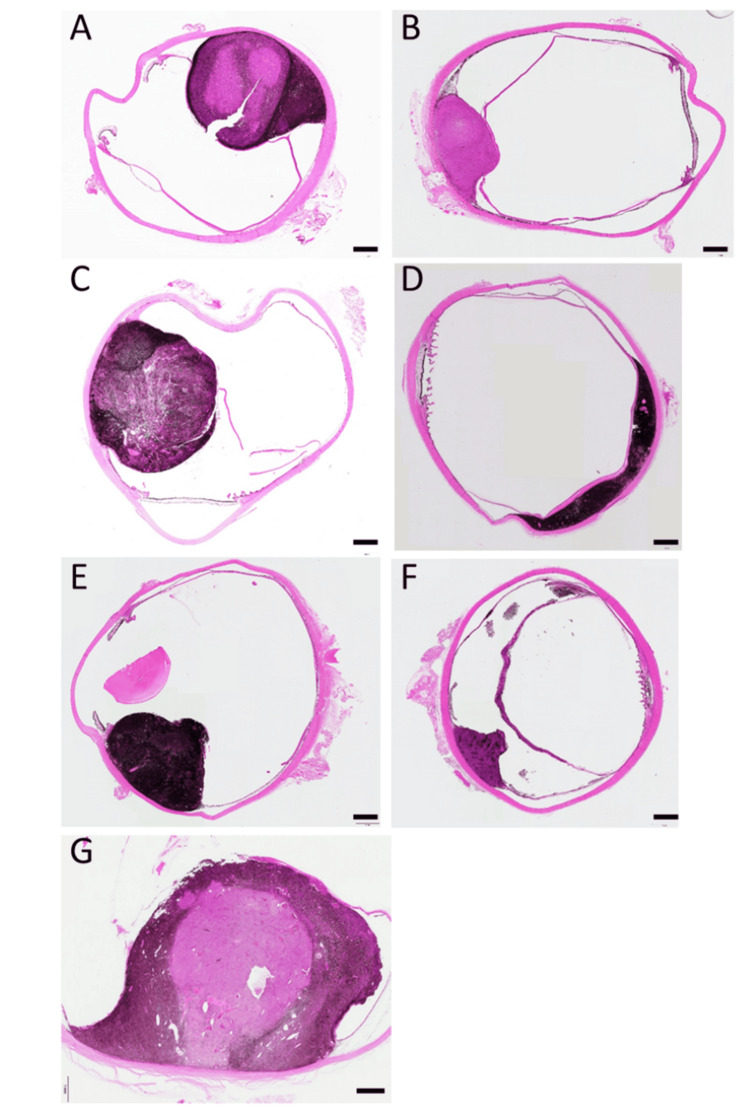
Hematoxylin-eosin-stained pathological sections of enucleated eyes with choroidal malignant melanoma. A: Case M1, B: Case M2, C: Case M3, D: Case M4, E: Case M5, F: Case M6, and G: Case M7. The clinical features of these cases are summarized in Table 1. Scale bar = 1 mm.

Whole-exome sequencing was performed in seven samples of retinoblastoma (Figure 1) and seven samples of choroidal malignant melanoma (Figure 2, Table 1). This study was approved as a retrospective genomic study by the Ethics Committee of Okayama University Graduate School of Medicine, Dentistry, and Pharmaceutical Sciences and Okayama University Hospital (approval number: 2101-003; approval date: February 12, 2021). The enucleated eyes were fixed in 10% neutral buffered formalin for 3 to 24 hours, cut in half, further fixed in formalin for 24 hours, and embedded in paraffin. The paraffin-embedded blocks were stored at room temperature in the Department of Pathological Diagnosis at Okayama University Hospital. The seven consecutive eyes with choroidal malignant melanoma were enucleated between 2008 and 2011, as reported previously [3]. The nine consecutive eyes with retinoblastoma were enucleated between 2006 and 2023. All patients had unilateral malignancy and showed no abnormalities in the fellow eye. One eye with retinoblastoma (R5) was excluded from this study because enucleation was performed long after systemic chemotherapy. Another eye with retinoblastoma (R8) was excluded because no intraocular tumor tissue remained in the paraffin sections.

**Table 1:** Clinical features of patients with retinoblastoma (R series) and choroidal malignant melanoma (M series). Case R5 (female) was excluded because the eye was enucleated after chemotherapy. Case R8 (male) was excluded because no tumor tissue remained in the preserved paraffin-embedded tissue block.

Case no.	Gender	Age at enucleation	Diagnosis	Laterality	Clinical picture	Pathological feature	Additional features
R1	Male	3 years 9 months	Retinoblastoma	Right eye	Nodular tumor	Rosette formation with marked mitosis	No chemotherapy
R2	Male	6 months	Retinoblastoma	Left eye	Nodular tumor	Rosette formation	No chemotherapy
R3	Male	3 years 4 months	Retinoblastoma	Right eye	Nodular tumor	Rosette formation	No chemotherapy
R4	Female	2 years 1 month	Retinoblastoma	Left eye	Nodular tumor	Fleurette formation with marked mitosis	No chemotherapy
R6	Male	1 year 7 months	Retinoblastoma	Left eye	Nodular tumor	Rosette formation	No chemotherapy
R7	Male	1 year 0 month	Retinoblastoma	Left eye	Nodular tumor	Rosette formation	Chemotherapy due to optic nerve infiltration
R9	Female	1 year 7 months	Retinoblastoma	Left eye	Nodular tumor	Diffuse proliferation	No chemotherapy
M1	Female	82 years	Malignant melanoma	Left eye	Nodular tumor	Epithelioid cell type with giant multinucleated cells	Liver metastasis
M2	Female	73 years	Malignant melanoma	Right eye	Nodular tumor	Epithelioid cell type	Right breast, head skin metastasis
M3	Male	80 years	Malignant melanoma	Left eye	Nodular tumor	Spindle type	No metastasis
M4	Male	60 years	Malignant melanoma	Left eye	Flatly infiltrating tumor	Epithelioid cell type with giant multinucleated cells	Liver metastasis
M5	Female	63 years	Malignant melanoma	Left eye	Nodular tumor	Epithelioid cell type	Liver metastasis
M6	Male	75 years	Malignant melanoma	Left eye	Flatly infiltrating tumor with small protrusion and vitreous seeding	Epithelioid cell type with vitreous seeding	No metastasis
M7	Male	58 years	Malignant melanoma	Left eye	Nodular tumor	Epithelioid cell type	No metastasis

Genomic DNA was extracted from 105 paraffin sections of 5 μm thickness and submitted to exome capture with the SureSelect XT Human All Exon V6 Kit (Agilent Technologies, Santa Clara, CA) and then to whole-exome sequencing using NovaSeq 6000 150-bp paired-end 7 Gb/sample (Illumina, San Diego, CA) at Macrogen Japan (Tokyo). In more detail, genomic DNA was isolated from paraffin sections using the Maxwell RSC DNA FFPE Kit (Promega Corporation, Madison, WI). The amount of genomic DNA was measured by a fluorescence-based quantification method (QuantiFluor dsDNA System, Promega Corporation) using the VICTOR Nivo Multimode Microplate Reader (PerkinElmer, Waltham, MA). Genomic DNA integrity was verified using the 4200 TapeStation System (Agilent Technologies) and expressed as the DNA integrity number (DIN), which determines the degree of fragmentation of a genomic DNA sample by measuring the distribution of signals of various sizes. The total amounts of DNA extracted from the paraffin sections ranged from 1.622 µg to 18.555 µg in seven retinoblastoma samples and from 0.669 µg to 4.562 µg in seven choroidal malignant melanoma samples (Table 2). The genomic DNA in all samples passed the quality requirements for library construction.

**Table 2:** Number of reads in each retinoblastoma sample (R series) and choroidal malignant melanoma sample (M series).

Sample no.	Total DNA amount extracted from paraffin sections	DNA integrity number (DIN)	DNA quality for library construction	Number of raw reads	Number of reads after quality control	Number of mapped reads	Number of reads after deduplication and recalibration
R1	4.426 μg	1.9	PASS	49,364,312	49,358,078	49,306,431	34,438,540
R2 (excluded)	1.622 μg	1.4	PASS	34,147,964	34,134,490	34,025,386	24,453,527
R3	2.636 μg	1.6	PASS	53,794,056	53,782,704	53,728,331	36,813,101
R4	2.811 μg	1.6	PASS	54,263,864	54,249,418	54,190,766	34,465,678
R6	2.793 μg	1.6	PASS	58,615,884	58,612,554	58,567,638	41,518,417
R7	18.555 μg	1.8	PASS	54,792,534	54,788,460	54,742,945	36,988,505
R9	10.948 μg	1.8	PASS	57,946,428	57,941,936	57,897,529	38,008,825
M1	4.562 μg	2	PASS	54,544,636	54,534,610	54,462,785	36,786,616
M2 (excluded)	0.682 μg	1.5	PASS	5,560,084	5,558,780	5,552,532	3,864,819
M3	1.594 μg	1.1	PASS	42,521,974	42,503,702	42,394,914	26,232,698
M4	0.831 μg	3.2	PASS	50,931,784	50,927,164	50,827,287	34,152,221
M5	0.744 μg	1	PASS	54,577,254	54,562,744	54,460,627	36,557,596
M6	0.669 μg	1.6	PASS	48,107,214	48,093,014	48,015,143	33,475,660
M7	2.592 μg	2.1	PASS	53,729,516	53,710,682	53,626,190	37,886,737

Quality control and mutation detection

**Figure 3:**
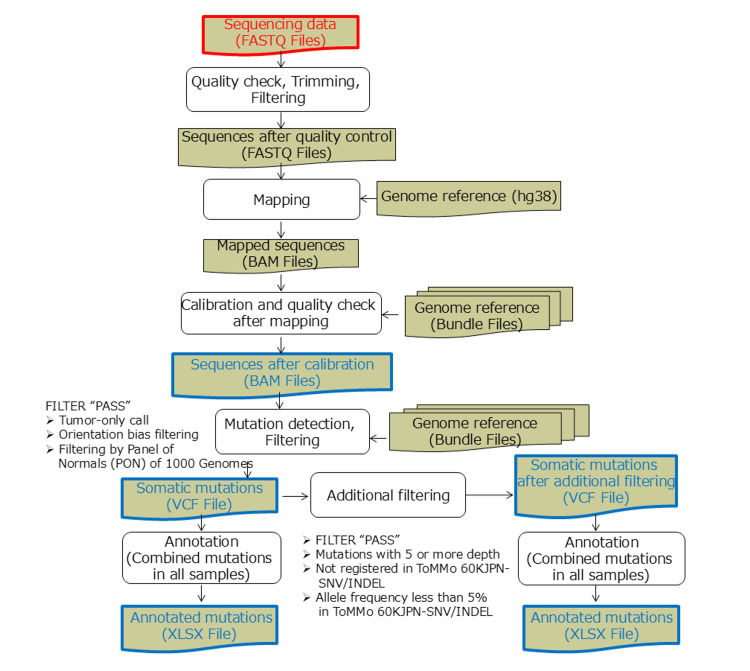
Flowchart for the detection of somatic mutations. Image created by Toshihiko Matsuo using Microsoft PowerPoint. BAM, Binary Alignment Map; VCF, Variant Call Format; XLSX, Microsoft Excel Open XML Spreadsheet

The sequencing data were subjected to quality control, trimming, and filtering. After quality control, the reads in the sequencing data were aligned to the reference genome sequence (hg38, GRCh38 Assembly, Genome Reference Consortium Human Build 38) (Figure 3). After the initial alignment, the mapped reads in Binary Alignment Map (BAM) files were processed to improve data quality, following the Genome Analysis Toolkit (GATK) Best Practices workflow (Broad Institute, https://gatk.broadinstitute.org/hc/en-us), including the exclusion of duplicated reads, local realignment, and adjustment of quality scores. Somatic mutations were called from tumor DNA sequences using GATK Mutect2 in tumor-only mode. The mutations were then filtered by a Panel of Normals (PON), which was constructed from 1000 Genomes samples. They were further filtered by the ToMMo 60KJPN-SNV/INDEL Allele Frequency Panel (v20240904) of the Tohoku Medical Megabank Project, Japan, to exclude presumed germline mutations. Annotations were assigned to the filtered mutations. To summarize, the detected somatic mutations were designated as “PASS” in the Mutect2 “Filter” field, had a read depth of 5 or greater at each mutation site, and either were not registered in the ToMMo 60KJPN-SNV/INDEL database or showed an allele frequency of less than 5% in the ToMMo 60KJPN-SNV/INDEL Allele Frequency Panel (v20240904). Two datasets of somatic mutations were prepared for retinoblastoma and choroidal malignant melanoma, respectively (Figure 3).

Driver mutation

**Figure 4:**
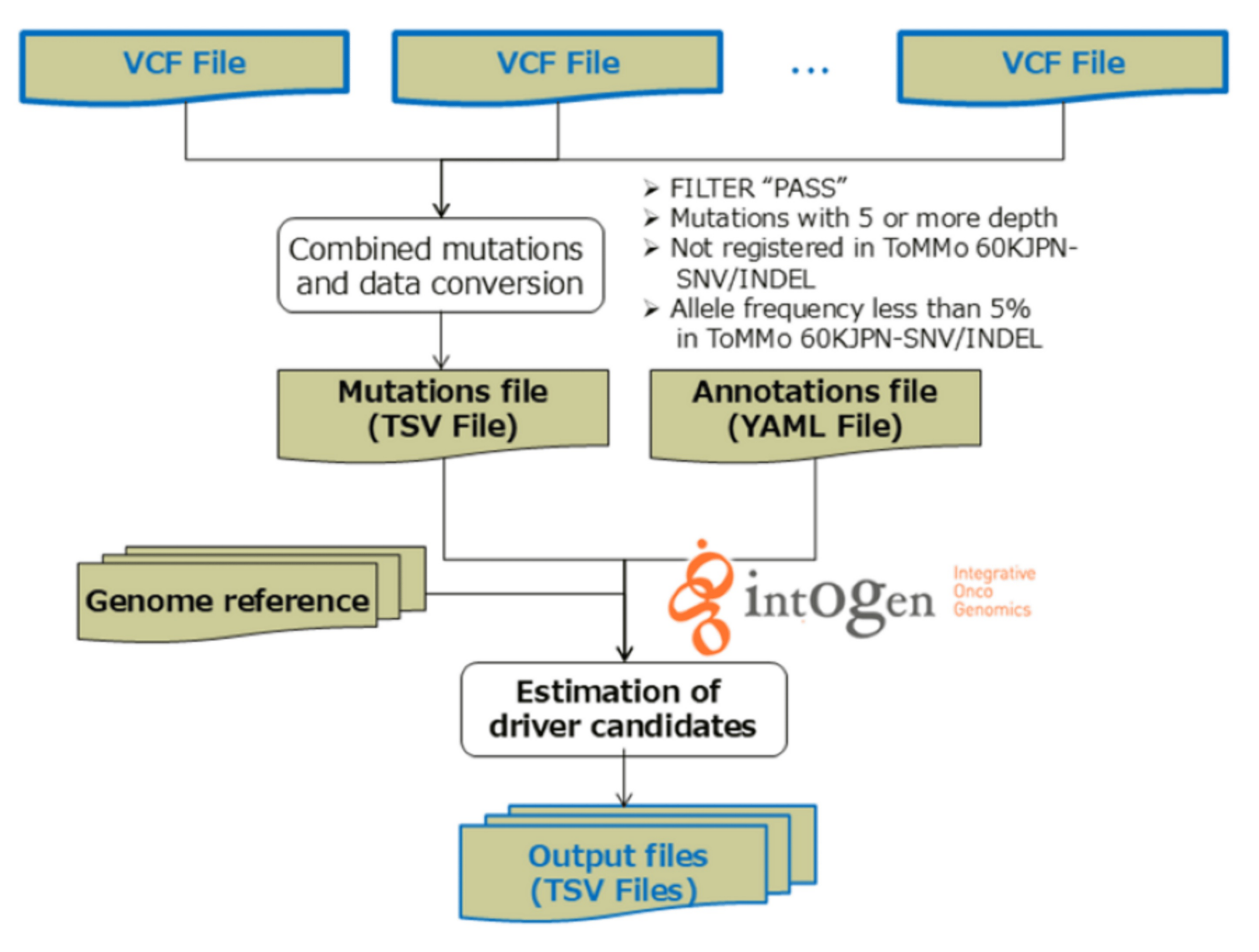
Flowchart for the detection of driver mutations. Image created by Toshihiko Matsuo using Microsoft PowerPoint. VCF, Variant Call Format; TSV, Tab-Separated Values; YAML, YAML (Yet Another Markup Language) Ain't Markup Language; IntOGen, Integrative OncoGenomics

Driver candidates in retinoblastoma or choroidal malignant melanoma were identified based on the significant accumulation of specific somatic mutations in specific genomic regions. Since it was difficult to predict a reliable background mutation rate in data from small sample sizes, an open database of Multi-Center Mutation Calling in Multiple Cancers (MC3) in The Cancer Genome Atlas (TCGA) was referenced to calculate the nucleotide context of three nucleotides (mutational signature), which was used as the background mutation model to predict driver candidates in small sample sizes. In addition, driver candidates were also estimated using Integrative OncoGenomics (IntOGen), a pipeline that combines the results of multiple driver-gene identification tools (Figure 4). The IntOGen pipeline was run using the default filtering criteria and significance thresholds. To identify driver genes, two distinct gene sets were extracted from the pipeline output. The first set comprised the final driver genes selected by the default filtering criteria (from drivers.tsv). The second set was a broader set that encompassed all candidate genes before the final filtering step (from unfiltered_drivers.tsv).

Enrichment analysis

Three lists of driver candidates in retinoblastoma or choroidal malignant melanoma were generated based on three criteria: (1) a gene with the same mutation shared by two or more samples (patients) that could influence gene function, (2) a recurrently mutated gene with three or more mutations that could influence gene function, and (3) a candidate gene identified before the final filtering step in driver mutation detection by IntOGen. Under criterion (2), in most cases, different kinds of mutations in a gene were present in three samples (patients), but on rare occasions, two or three different kinds of mutations were present in a single gene within the same sample. The genes detected under each criterion or under any of the three criteria were used to identify specific pathways in which mutated genes were accumulated. Functional enrichment analysis was performed using the clusterProfiler and ReactomePA packages. As pathway databases, four open databases were used: Gene Ontology (GO), Kyoto Encyclopedia of Genes and Genomes (KEGG), the Hallmark Gene Set of the Molecular Signatures Database (MSigDB), and Reactome. Statistical significance was assessed by the false discovery rate (FDR), and pathways with a *q*-value ≤ 0.05 were considered significantly enriched. The software used in this study is listed in Table 3 [25,26,27,28,29,30,31,32,33,34,35,36,37,38,39,40,41,42].

**Table 3:** List of software used in this study.

Software	Version	Purpose	References
FASTQ	0.23.4	Quality control	[25]
BWA	0.7.17	Mapping	[26,27]
GATK	4.1.2.0	Quality control, calibration	[28-31]
Mutect2	4.1.2.0	SNV/indel detection, filtering	[28-32]
ANNOVAR	2020/06/07	Annotation	[33]
OncodriveCLUSTL	1.1.3	Driver candidates	[34]
IntOGen	2024.11 (2.1.1)	Driver candidates	[35]
dNdScv	0.0.1.0	Driver candidates	[36]
CbaSE	1.1	Driver candidates	[37]
MutPanning	2.0	Driver candidates	[38]
HotMAPS	1.1.3	Driver candidates	[39]
smRegions	0.1	Driver candidates	[35]
OncodriveFML	2.4.0	Driver candidates	[40]
clusterProfiler	4.6.2	Enrichment analysis	[41]
ReactomePA	1.42.0	Enrichment analysis	[42]

## Results

Reads in the sequencing data of each sample were of high quality. Table 2 shows the results of quality control after the trimming of sequencing adapters and conventional processing. The total output after the trimming of sequencing adapters ranged from 5 to 9 Gb in all samples except for sample M2, and almost all reads were mapped to the reference genome sequence (hg38). However, the total output was reduced to about two-thirds when the reads were limited to those mapped to the targeted exome regions. The SureSelect Human All Exon V6 Kit was designed to cover genomic regions of 60,507,855 bp, based on targeted exome regions of 38,227,700 bp. The results indicated that many reads were mapped to regions outside the targeted exomes. Based on the total output, one sample with the lowest output (R2) in retinoblastoma and another sample with the lowest output (M2) in choroidal malignant melanoma were excluded from driver mutation analysis and pathway enrichment analysis (Table 2).

Table 4 shows the duplication rates and the range of depth in each sample. The duplication rates in retinoblastoma samples, after the exclusion of sample R2, ranged from 16% to 33%, with a median of 23%, which was comparable to the empirical duplication rate of about 20%. In contrast, the duplication rates in choroidal malignant melanoma samples, after the exclusion of sample M2, were higher, ranging from 33% to 53%. The mean depth in retinoblastoma samples ranged from 25 to 43, whereas the mean depth in choroidal malignant melanoma samples was lower, ranging from 13 to 28.

**Table 4:** Total output, duplication rate, and depth in each sample of retinoblastoma (R series) and choroidal malignant melanoma (M series).

Sample No.	Total output (Gb)	Duplication rate	Mean depth	Ratio of depth ≥1	Ratio of depth ≥2	Ratio of depth ≥10	Ratio of depth ≥20	Ratio of depth ≥30	Ratio of depth ≥40
R1	2.918	0.3377	25.63	0.9412	0.9328	0.7944	0.5519	0.3449	0.2011
R2 (excluded)	1.008	0.6794	8.589	0.9115	0.8577	0.3937	0.0683	0.0061	0.0008
R3	3.6	0.2576	31.57	0.9427	0.9369	0.8525	0.6647	0.4583	0.2911
R4	3.503	0.2426	31.64	0.9438	0.9387	0.8611	0.6669	0.4533	0.2872
R6	4.367	0.2338	38.37	0.9377	0.9278	0.8417	0.7034	0.5497	0.4061
R7	4.446	0.169	43.36	0.9456	0.9392	0.8637	0.7338	0.5863	0.4483
R9	4.508	0.1784	43.99	0.9462	0.9421	0.8916	0.7785	0.6265	0.4728
M1	3.196	0.3323	28.63	0.9403	0.9299	0.7422	0.5259	0.3766	0.2632
M2 (excluded)	0.1941	0.4973	1.739	0.6285	0.4051	0.0103	0.0003	0	0
M3	1.593	0.4605	13.08	0.9013	0.838	0.4229	0.2266	0.1239	0.0677
M4	2.161	0.4956	19.29	0.9391	0.9259	0.6919	0.4102	0.2247	0.1067
M5	2.018	0.5359	16.96	0.9284	0.9016	0.5567	0.3282	0.1918	0.1044
M6	2.293	0.4941	20.59	0.9441	0.9357	0.7532	0.4555	0.235	0.106
M7	2.617	0.3856	23.48	0.9283	0.9048	0.6269	0.4116	0.292	0.2041

Table 5 shows the number of somatic mutations detected in each sample. The Mutect2-PASS columns show the numbers of total mutations, single-nucleotide variants, insertions and deletions (indels), and multiple-nucleotide variants after orientation-bias filtering and the exclusion of variants reported in the 1000 Genomes Project. The numbers of mutations in each category remained essentially the same after additional filtering, which included a depth of 5 or greater for each mutation and no registration or an allele frequency of less than 5% in the ToMMo 60KJPN-SNV/INDEL Allele Frequency Panel (v20240904) of the Tohoku Medical Megabank Project, Japan.

**Table 5:** The number of somatic mutations in each retinoblastoma sample (R series) and choroidal malignant melanoma sample (M series). Additional filtering indicates a depth of 5 or greater for each mutation and no registration or an allele frequency of less than 5% in the Tohoku Medical Megabank of Japan (ToMMo 60KJPN-SNV/INDEL Allele Frequency Panel). SNV, single-nucleotide variant; Indel, insertion and deletion; MNV, multiple-nucleotide variant

Sample No.	Mutect2-PASS				Additional filtering			
	Total variants	SNV	Indel	MNV	Total variants	SNV	Indel	MNV
R1	2,521	1,103	864	554	2,469	1,074	851	544
R2 (excluded)	2,335	1,463	402	470	1,971	1,255	349	367
R3	766	554	75	137	745	545	71	129
R4	718	594	24	100	693	573	22	98
R6	470	416	19	35	450	400	17	33
R7	339	322	11	6	318	306	6	6
R9	319	297	11	11	308	289	8	11
M1	3,778	1,475	1,436	867	3,687	1,445	1,409	833
M2 (excluded)	22,777	21,712	618	447	2,274	1,952	187	135
M3	4,597	2,142	1,808	647	4,254	2,017	1,674	563
M4	3,253	1,323	1,010	920	3,169	1,294	988	887
M5	4,970	1,931	2,252	787	4,837	1,889	2,198	750
M6	1,504	694	491	319	1,463	679	480	304
M7	4,209	1,294	1,933	982	4,028	1,245	1,866	917

After the additional filtering and the exclusion of one low-quality sample from each group, a list of somatic mutations was prepared for six samples of either retinoblastoma or choroidal malignant melanoma. Mutations that could influence gene function were extracted from the list of genes based on the annotation. The number of genes with the same mutation shared by two or more samples (patients) that could influence gene function was 22 in retinoblastoma samples and 36 in choroidal malignant melanoma samples (Table 6). The number of recurrently mutated genes with three or more mutations that could influence gene function was 63 in retinoblastoma samples and 1061 in choroidal malignant melanoma samples (Table 6).

**Table 6:** Genes with somatic mutations in retinoblastoma and choroidal malignant melanoma.

	Retinoblastoma	Choroidal malignant melanoma
Genes with the same mutation shared by two or more samples	SLC9C2, ENAH, CFAP65, ABCB6, DNAH1, FLNB, CUL7, TINAG, RASGRP2, C11orf65, SYNE2, UBAP1L, ZNF598, GP1BA, ENDOV, FOXK2, ZNF507, TDRD12, FAAP24, WDR87, CAPN12, LAMA5	ATP13A2, OMA1, MYBPHL, LY9, ADAMTS4, RGS18, MFSD9, RAPGEF4, ATG9A, CPNE9, EAF1, DKK2, POLK, RAB44, ITPRID1, CRYGN, EBF2, IKBKB, SCRT1, VPS11, GRIN2B, ENDOU, RAPGEF3, ACACB, MAP4K5, TRIP11, PLA2G4F, MYEF2, PKD1, MOCOS, MBD3, GNA11, ZNF773, SUSD2, CELSR1, PASD1
Recurrently mutated genes three or more times	ADAM29, ADGRV1, ANKLE1, CD79A, CDH23, CFAP54, CFAP65, CMYA5, CNTNAP2, CNTRL, COL6A3, DLGAP5, DNAH11, DOCK5, DOCK7, DYNC2H1, EIF3A, EML6, F3, FBXO22, FLNB, FOCAD, FOXK2, FRAS1, FREM1, FRYL, HERC1, ITSN2, JAKMIP3, KMT2B, LAMA5, LAMC3, LCA5L, LCOR, LMBRD2, LRP1, LRP5, MRC1, MUC19, NBEAL2, NHS, OBSCN, PDZD8, PIEZO1, PKD1L2, RAD18, RXRA, SAMD9, SPG11, SRRM2, STARD9, STIL, SYNE1, SYNE2, TENM4, TINAG, TRPM4, TRPV3, TRRAP, TTC3, TTN, USP34, WDFY4	1,061 genes

As for driver mutations (Table 7), 30 genes were detected in retinoblastoma samples, while none were detected in choroidal malignant melanoma samples at the significant threshold level of Q < 0.05. When the threshold level was loosened to Q < 0.10, 31 genes were detected as driver candidates in retinoblastoma, and two genes (*SCRT1* and *GNA11*) were detected in choroidal malignant melanoma. In the estimation of driver genes by IntOGen, no drivers were detected in retinoblastoma samples (Table 7). In contrast, eight genes were detected as drivers in choroidal malignant melanoma, and four (*GNA11*, *LRP1B*, *BRCA2*, and *FAT1*) of these eight genes were registered as top 20 genes in the list for choroidal malignant melanoma in the Cancer Gene Census (CGC) database of the Catalogue of Somatic Mutations in Cancer (COSMIC) (Table 8). In retinoblastoma, the most famous driver gene, *RB1*, which was predominantly listed as 120/302 in the CGC database of COSMIC, was found in one (R7) of 6 samples, in addition to the excluded sample (R2), in the present study.

**Table 7:** Lists of genes with driver mutations in retinoblastoma and choroidal malignant melanoma. IntOGen, Integrative OncoGenomics.

Methods	Retinoblastoma	Choroidal malignant melanoma
OncodriveCLUSTL driver candidates (Q<0.05)	CAPN12, LY75-CD302, LY75, SRY, PHOX2B, ZNF598, DNAH1, RASGRP2, FLNB, CUL7, UBAP1L, FOXK2, ABCB6, SRCAP, PWP1, ENDOV, FAAP24, SYNE2, LAMA5, ENAH, TINAG, WDR87, ZNF507, GP1BA, CFAP65, TDRD12, PLP1, DKK3, C11orf65, SLC9C2	None
OncodriveCLUSTL driver candidates (Q<0.1)	CAPN12, LY75-CD302, LY75, SRY, PHOX2B, ZNF598, DNAH1, RASGRP2, FLNB, CUL7, UBAP1L, FOXK2, ABCB6, SRCAP, PWP1, ENDOV, FAAP24, SYNE2, LAMA5, ENAH, TINAG, WDR87, ZNF507, GP1BA, CFAP65, TDRD12, PLP1, DKK3, C11orf65, SLC9C2, HEATR4	SCRT1, GNA11
IntOGen-filtered drivers	None	BRCA2, FAT1, FAT3, GNA11, IKBKB, KMT2C, LRP1B, PRDM1
IntOGen-filtered driver candidates	A4GNT, ABCB6, ADAMTS9, ADAT1, ANKLE1, C11orf65, CAMSAP1, CAPN12, CFAP65, CMYA5, COL6A3, DKK3, DNAH11, FAAP24, FOCAD, GP1BA, HAPLN3, KLHL17, LY75, NBEAL2, PHOX2B, PIEZO1, PLP1, PPP1R26, PWP1, RXRA, SAMD9, SELENBP1, SLC9C2, SRCAP, SRRM2, SRY, SYNE1, SYT12, TINAG, TRPM4, TRPV3, TRRAP, TTC3, WDR87, ZNF507	ADAMTS4, ADCK2, ALDH16A1, ALPK2, ANGPT1, ATP13A2, BOP1, BRCA2, CACNA1H, CAMSAP1, CASKIN1, CCDC38, CCDC73, CELSR1, CEP97, CFAP206, COL18A1, COL4A2, CPAMD8, CRAMP1, DAGLB, DCHS2, DDX54, DKK2, DNAAF1, DNAH12, DSCAM, EAF1, EBAG9, ECE2, ECT2, EFCAB5, EFCAB6, EIF2B2, ENDOU, EP300, EP400, EXOC2, FANCL, FAT1, FAT2, FAT3, FAT4, FBXO47, FCRL5, FGB, FNDC3B, FRY, GNA11, GPR21, HADHB, HELZ2, HIPK2, HMCN2, IKBKB, INPP4A, ITPRID1, JMJD1C, KIF26A, KIF4B, KLF2, KMT2C, LAMA3, LMNB1, LRP1, LRP1B, LRP2BP, LRRC15, LRRC23, LTBP1, LY9, MAP4K5, MBD3, MDN1, MOCOS, MSH4, MYEF2, MYO16, MYO9B, NBEAL2, NDUFB7, NEB, NFATC1, NOX5, NRP2, NTN1, NUP210, OBSCN, PASD1, PCDH19, PDZD2, PKD1, PKHD1, PLA2G4F, POLK, PRDM1, PROCA1, RAB44, RALGAPA2, RAP1GAP2, RAPGEF3, RAPGEF4, RELT, RIC1, RIMS1, RNF214, RYR1, SAMD9, SCRT1, SDK1, SEC31A, SGSM2, SH3RF3, SHANK2, SNRNP200, SOGA3, SPTBN1, SUSD2, SVEP1, TACC2, TAPBPL, TCEAL8, TLN2, TP53BP2, TRAK1, TRIP11, TTN, ULK1, UNC13A, USH2A, VPS11, VPS13B, VWF, WDFY3, YEATS2, ZNF773

**Table 8:** Top 20 genes for choroidal malignant melanoma in the Cancer Gene Census (CGC) database of the Catalogue of Somatic Mutations in Cancer (COSMIC).

Gene name	COSMIC	This study	Cases in this study	IntOGen
GNAQ	640/1861	0/6	None	
GNA11	569/1593	2/6	M1, M3	PASS
BAP1	339/985	1/6	M1	
SF3B1	184/1052	1/6	M6	
BRAF	112/1574	0/6	None	
NRAS	34/1035	0/6	None	
LRP1B	25/378	3/6	M1, M4, M7	PASS
KIT	20/844	2/6	M5, M7	
FGFR4	16/427	1/6	M3	
CHEK2	14/335	1/6	M6	
PTEN	13/538	0/6	None	
BRCA2	13/426	4/6	M3, M5, M6, M7	PASS
DICER1	13/336	3/6	M1, M3, M6	
FBXW7	11/485	1/6	M7	
ATM	10/434	1/6	M4	
PDE4DIP	10/218	0/6	None	
KMT2D	9/335	3/6	M1, M3, M5	
TSC2	9/335	2/6	M4, M7	
FAT1	9/335	1/6	M7	PASS
TP53	8/501	0/6	None	

**Figure 5:**
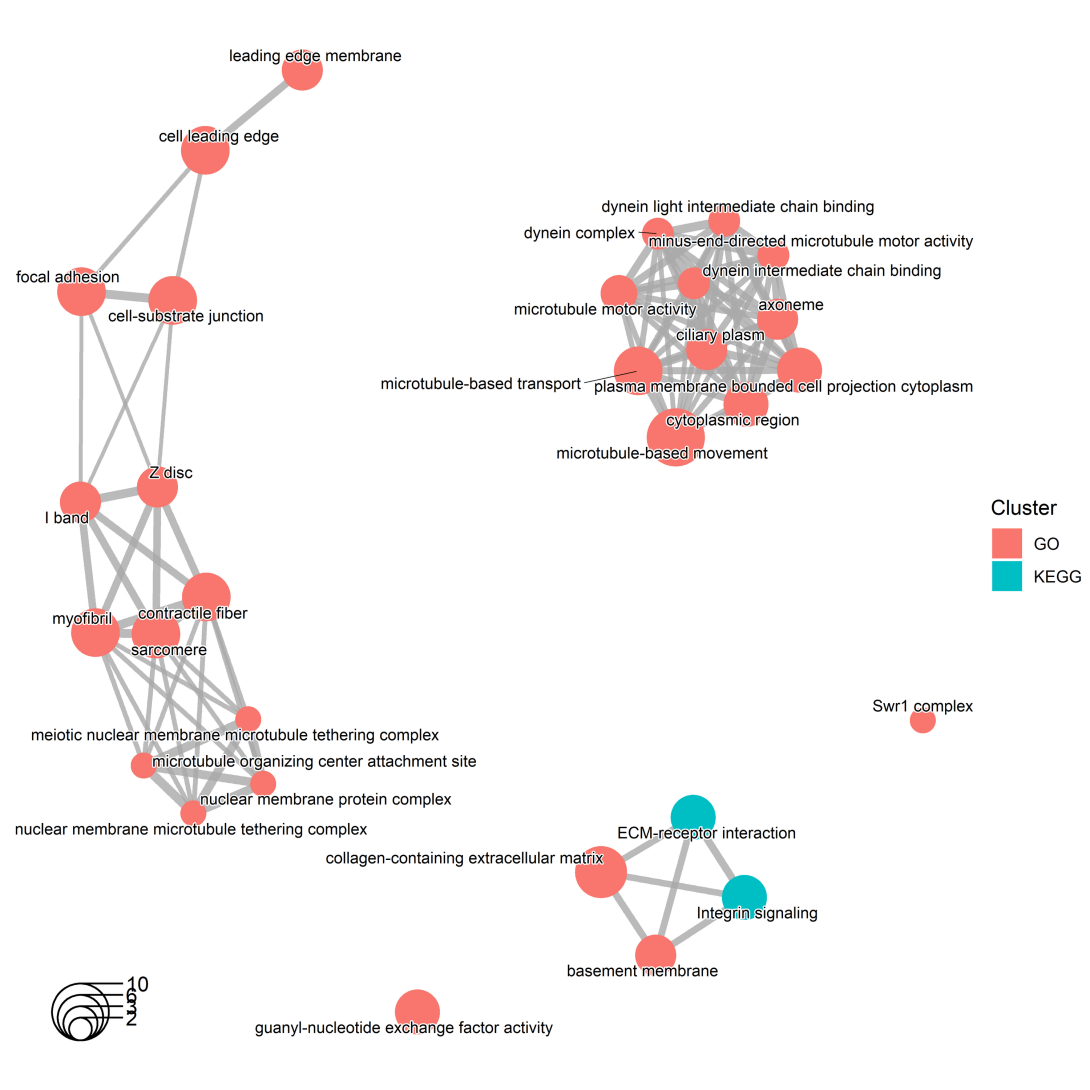
Pathway enrichment map for retinoblastoma. Pathway enrichment map for retinoblastoma based on the combination of the Gene Ontology (GO) and Kyoto Encyclopedia of Genes and Genomes (KEGG) databases. Image created by the authors using enrichplot (R version 4.2.0, R Foundation for Statistical Computing, Vienna, Austria).

**Figure 6:**
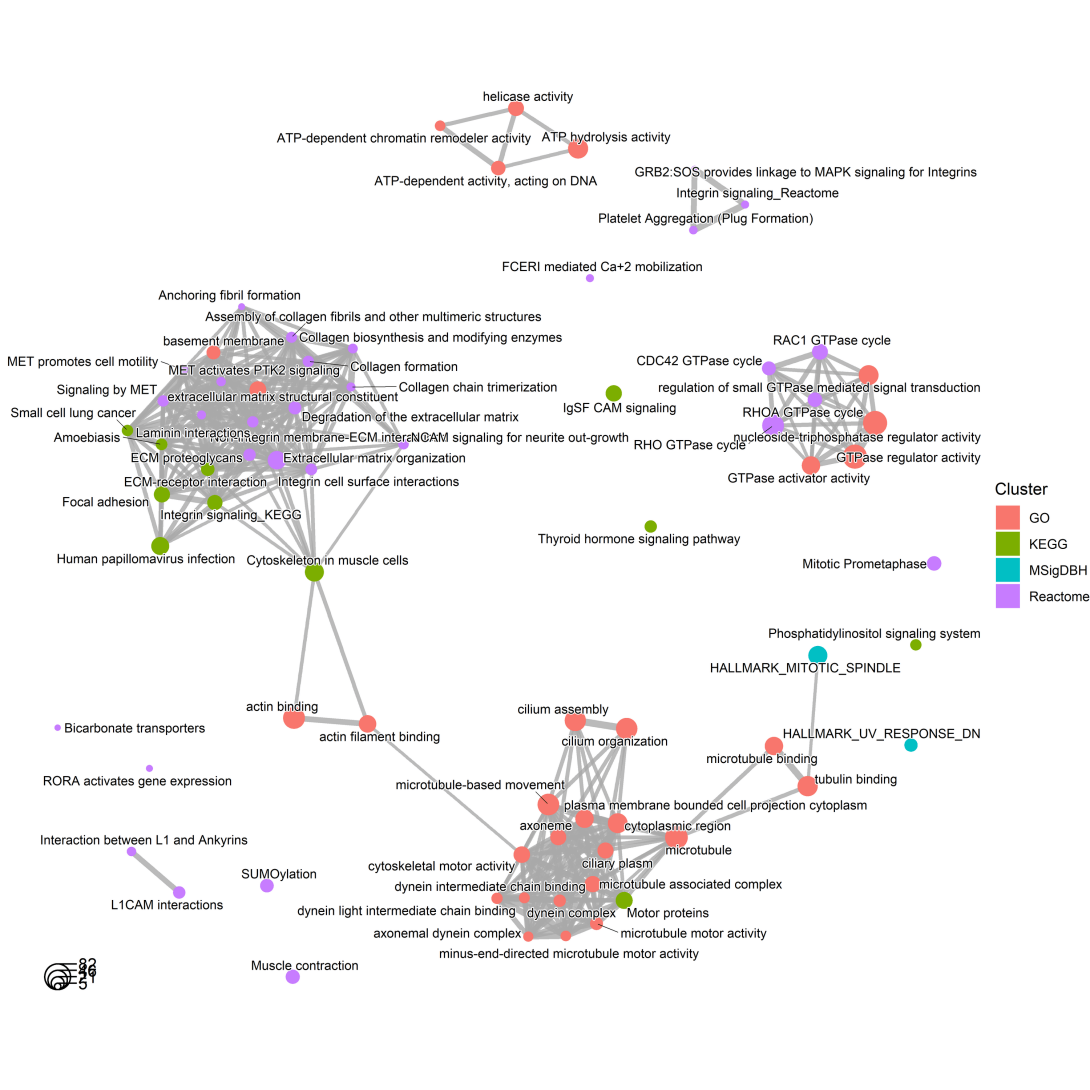
Pathway enrichment map for choroidal malignant melanoma. Pathway enrichment map for choroidal malignant melanoma based on the combination of the Gene Ontology (GO), Kyoto Encyclopedia of Genes and Genomes (KEGG), Hallmark Gene Set of the Molecular Signatures Database (MSigDBH), and Reactome databases. Only the top 30 genes for GO and Reactome are included (Table 9). Image created by the authors using enrichplot (R version 4.2.0, R Foundation for Statistical Computing, Vienna, Austria).

Table 9 shows the number of pathways detected by different kinds of enrichment analyses in the databases of GO, KEGG, MSigDB, and Reactome. Figure 5 shows enrichment maps in combination [43], depicted with 28 genes detected in GO and 2 genes detected in KEGG for retinoblastoma. Figure 6 shows enrichment maps in combination [43], depicted with the top 30 genes detected in all four databases: GO, KEGG, the hallmark gene set of MSigDB, and Reactome.

**Table 9:** Number of pathways in enrichment analyses using genes with somatic mutations in retinoblastoma and choroidal malignant melanoma. GO, Gene Ontology (MF: molecular function, CC: cellular component, BP: biological process); KEGG, Kyoto Encyclopedia of Genes and Genomes (1: metabolism, 2: genetic information processing, 3: environmental information processing, 4: cellular processes, 5: organismal systems, 6: human diseases, 7: drug development); MSigDB, the Hallmark Gene Set of the Molecular Signatures Database; IntOGen, Integrative OncoGenomics

Dataset	Candidate genes that influence the function	GO	KEGG	MSigDB	Reactome
Retinoblastoma	A: Genes with the same mutation shared by two or more samples	0	0	0	1
	B: Recurrently mutated genes three or more times	22	4	0	0
	C: IntOGen-filtered driver candidates	1	0	0	3
	A, B, or C	28	2	0	0
Choroidal malignant melanoma	A: Genes with the same mutation shared by 2 samples or more	0	0	0	3
	B: Recurrently mutated genes 3 times or over	410	12	2	48
	C: IntOGen-filtered driver candidates	12	0	0	3
	A, B, or C	385	12	2	47

## Discussion

The aim of this pilot study is to test whether whole-exome sequencing would be applicable to formalin-fixed, paraffin-embedded malignant tissues of enucleated eyes. Intraocular tumors are extremely rare and tiny in size because of the limited space within the eyeball. In the case of extirpation of the primary lesion, whole-eye extirpation, namely eye enucleation, is usually performed since partial resection of the eyeball is technically difficult and cannot preserve visual function. The two predominant intraocular malignancies are retinoblastoma, which arises from the neurosensory retinal tissue, and choroidal malignant melanoma, which arises from choroidal melanocytes. Unlike other malignancies that occur at other sites in the body, the normal tissues corresponding to retinoblastoma or choroidal malignant melanoma are extremely limited in amount. These situations, which are specific to intraocular malignancies, hinder standard paired analysis to extract malignancy-specific mutations by comparison with the normal counterpart and thus present a major limitation in cancer genome analysis.

The second limitation is the process of formalin fixation after eye enucleation. The intraocular tumors expand into the vitreous, which is a large extracellular space within the confines of the collagen-based hard outer shell of the eyeball, called the sclera. The enucleated eyes are immersed in 10% neutral buffered formalin solution, as recommended for cancer genome analysis, and are usually cut into halves on the following day to further fix the intraocular tissues. Overall, the fixation period is within 72 hours, as recommended. However, the intraocular tissues might not be well fixed since the sclera is a barrier to formalin penetration into the intraocular space. The presence of the large extracellular space of the vitreous might lead to deformation of the intraocular tissues when the eyeball is cut into halves.

To summarize the above discussion, this study had three major limitations that were apparent from the start: (1) tumor tissue-only data with no data from the normal counterpart, (2) a small amount of tumor tissue, and (3) formalin-fixed, paraffin-embedded tissue with a risk of delayed fixation of the intraocular tissue. Keeping these limitations in mind, we conducted the present study as a pilot study with small sample sizes. Due to the small volume of the intraocular tumors, 105 paraffin sections were used to isolate the tumor areas, and genomic DNA was extracted. As expected, the amount of genomic DNA was rather small (Table 2), but the DNA quality was sufficient to proceed to whole-exome sequencing. One of seven samples of retinoblastoma and one of seven samples of choroidal malignant melanoma were excluded from further analyses based on the smallest number of reads in each group (Table 2). In the two excluded samples, one for retinoblastoma (R2) and one for choroidal malignant melanoma (M2), the mean depth of reads was apparently lower than that in the other six samples in each group (Table 4). These findings support the appropriateness of the exclusion criteria, also from the viewpoint of mean read depth. It should still be noted that the overall lower read depth in each sample had the potential to generate false-positive somatic variants and thus reduce statistical power as a methodological constraint.

Two analytical methods used in this study were (1) the detection of driver genes or candidates and (2) pathway enrichment analysis. Driver genes are key genes with mutations that play an essential role in dysregulated cell proliferation and survival, leading to carcinogenesis. Other genes with mutations that might not be involved in carcinogenesis are called passenger genes. Several software programs have been developed to detect driver genes (Table 3), and in this study, we adopted a widely used program, OncodriveCLUSTL [34], and also used IntOGen, which combines the results from different driver-gene prediction programs [35]. In the analysis by IntOGen, no driver genes were detected in retinoblastoma, whereas 8 genes were detected in choroidal malignant melanoma. The present study appears to function correctly in detecting genes with somatic mutations since 4 of the 8 driver genes detected in choroidal malignant melanoma are included in the list of the top 20 genes for choroidal malignant melanoma in the open-access Cancer Gene Census (CGC) database of the Catalogue Of Somatic Mutations In Cancer (COSMIC) [44,45,46].

In pathway enrichment analyses, we designed three categories of genes with somatic mutations: (1) genes with the same mutation shared by two or more samples, (2) recurrently mutated genes with three or more mutations, and (3) genes identified as IntOGen-filtered driver candidates. Then, the genes with somatic mutations in any of the three categories were combined and used as input for four databases: GO, KEGG, MSigDB, and Reactome. In the enrichment map, three major clusters were detected in retinoblastoma, and three major clusters were detected in choroidal malignant melanoma. For instance, dynein would be involved in retinoblastoma [47], and *MET* would be involved in choroidal malignant melanoma [48]. The process of the analyses in the present study suggests that the combination of different software programs would lead to more reliable results, even under conditions in which whole-exome sequencing results were not of optimal quality. The study, however, should be considered preliminary and hypothesis-generating research that may provide a foundation for future larger-scale investigations.

## Conclusions

Whole-exome sequencing was applied to formalin-fixed, paraffin-embedded enucleated eyes with retinoblastoma and choroidal malignant melanoma in a pilot study. Genomic DNA was extracted from intraocular tumor areas in 105 paraffin sections of 5 µm thickness from seven samples of retinoblastoma and seven samples of choroidal malignant melanoma and submitted to the whole-exome sequencing process. One of the seven samples of retinoblastoma and one of the seven samples of choroidal malignant melanoma were excluded from the study based on lower total output and lower read depth compared with the other samples in each group.

Two analytical methods used in this study were (1) the detection of driver genes or candidates and (2) pathway enrichment analyses. Genes with somatic mutations (single-nucleotide variants, insertions and deletions, and multiple-nucleotide variants) were screened for driver genes and driver candidates by different programs to search for driver mutations, and the results were combined by the IntOGen program. For pathway enrichment analyses, candidate genes with somatic mutations were first selected by three criteria: genes with the same mutation shared by two or more samples, recurrently mutated genes with three or more mutations, and genes of driver candidates identified by combining several different driver-mutation-detecting programs in IntOGen. Using candidate genes detected by any of the three criteria as input, enrichment analyses were performed in retinoblastoma and choroidal malignant melanoma using four open databases: GO, KEGG, MSigDB, and Reactome. The enrichment maps showed three major pathways that differed between retinoblastoma and choroidal malignant melanoma. From the beginning of this study, it was evident that there would be limitations related to the small amounts of DNA obtained from formalin-fixed, paraffin-embedded small-sized tissues with no normal counterpart tissue as a control. Consequently, whole-exome sequencing resulted in lower read depth in each sample and had the potential for false-positive somatic variants and thus reduced statistical power. Even with these limitations, this exploratory and preliminary study provided clues to somatic mutations that were enriched in certain pathways and differed between retinoblastoma and choroidal malignant melanoma.
